# Comparative Genome Analysis and Global Phylogeny of the Toxin Variant Clostridium difficile PCR Ribotype 017 Reveals the Evolution of Two Independent Sublineages

**DOI:** 10.1128/JCM.01296-16

**Published:** 2017-02-22

**Authors:** M. D. Cairns, M. D. Preston, C. L. Hall, D. N. Gerding, P. M. Hawkey, H. Kato, H. Kim, E. J. Kuijper, T. D. Lawley, H. Pituch, S. Reid, B. Kullin, T. V. Riley, K. Solomon, P. J. Tsai, J. S. Weese, R. A. Stabler, B. W. Wren

**Affiliations:** a Department of Pathogen Molecular Biology, London School of Hygiene and Tropical Medicine, London, United Kingdom; b UCL Centre for Clinical Microbiology, University College London, Royal Free Campus, London, United Kingdom; c Public Health Laboratory London, Division of Infection, The Royal London Hospital, London, United Kingdom; d National Institute for Biological Standards and Control, South Mimms, United Kingdom; e Edward Hines, Jr., Veterans Affairs Hospital, Hines, Illinois, USA; f Institute of Microbiology and Infection, University of Birmingham, Edgbaston Campus, Birmingham, United Kingdom; g Public Health England (PHE), Public Health Laboratory Birmingham (PHLB), Birmingham Heartlands Hospital, Heart of England NHS Foundation Trust, Bordesley Green East, Birmingham, United Kingdom; h Department of Bacteriology II, National Institute of Infectious Diseases, Tokyo, Japan; i Department of Laboratory Medicine and Research Institute of Bacterial Resistance, Yonsei University College of Medicine, Seoul, South Korea; j National Reference Laboratory for Clostridium difficile, Leiden University Medical Centre and RIVM, Bilthoven, The Netherlands; k Wellcome Trust Sanger Institute, Wellcome Trust Genome Campus, Hinxton, Cambridgeshire, United Kingdom; l Department of Medical Microbiology, Medical University of Warsaw, Warsaw, Poland; m Department of Molecular and Cell Biology, University of Cape Town, Rondebosch, South Africa; n The University of Western Australia, School of Pathology and Laboratory Medicine, Crawley, Australia; o School of Medicine and Medical Science, UCD Veterinary Sciences Centre, University College Dublin, Belfield, Dublin, Ireland; p University of Exeter, Bioscience, College of Life and Environmental Science, Exeter, United Kingdom; q Department of Medical Laboratory Science and Biotechnology, National Cheng Kung University, Medical College, Tainan, Taiwan; r Department of Pathobiology, University of Guelph, Guelph, Ontario, Canada; s Loyola University Chicago Stritch School of Medicine, Maywood, Illinois, USA; t Center of Infectious Disease and Signaling Research, National Cheng Kung University, Tainan, Taiwan; The Johns Hopkins University School of Medicine

**Keywords:** Clostridium difficile, sequencing, SNPs, ribotype 017, evolution, phylogenetics, antibiotic resistance, phylogeny

## Abstract

The diarrheal pathogen Clostridium difficile consists of at least six distinct evolutionary lineages. The RT017 lineage is anomalous, as strains only express toxin B, compared to strains from other lineages that produce toxins A and B and, occasionally, binary toxin. Historically, RT017 initially was reported in Asia but now has been reported worldwide. We used whole-genome sequencing and phylogenetic analysis to investigate the patterns of global spread and population structure of 277 RT017 isolates from animal and human origins from six continents, isolated between 1990 and 2013. We reveal two distinct evenly split sublineages (SL1 and SL2) of C. difficile RT017 that contain multiple independent clonal expansions. All 24 animal isolates were contained within SL1 along with human isolates, suggesting potential transmission between animals and humans. Genetic analyses revealed an overrepresentation of antibiotic resistance genes. Phylogeographic analyses show a North American origin for RT017, as has been found for the recently emerged epidemic RT027 lineage. Despite having only one toxin, RT017 strains have evolved in parallel from at least two independent sources and can readily transmit between continents.

## INTRODUCTION

Clostridium difficile is a spore-forming obligate anaerobe that continues to be the leading cause of health care-associated infections in the developed world (1, 2). There are six main lineages that broadly split into PCR ribotypes (RTs) associated with RT027, RT023, RT017, RT078, a grouping of diverse RTs, and the recently identified novel lineage containing RT131 (3). The global emergence of the RT027 strain was responsible for multiple outbreaks and increased disease severity in Canada and the United States in 2001 (4). This strain has since spread to South America (5–7), China (8), Japan (9), Hong Kong (10), South Korea (11, 12), Taiwan (13), Singapore (14), Australia (15, 16), Saudi Arabia (17), Israel (18), New Zealand (19), and throughout Europe (5, 20–28). Although RT027 remains the dominant clone in the United States, Europe has seen a decline in RT027 with a simultaneous increase in other virulent RTs, such as RT017 and RT078 (29).

Using whole-genome sequencing (WGS) and phylogenetic analysis, He et al. (4) identified the presence of two genetically distinct sublineages of RT027 through single-nucleotide polymorphism (SNP) analysis; both had emerged in North America within a relatively short period after acquiring the same fluoroquinolone resistance-conferring mutation containing an alteration in *gyrA* and a highly related conjugative transposon (4). The two epidemic sublineages showed distinct patterns of global spread, with one lineage spreading more widely and causing health care-associated outbreaks globally (4).

Traditionally, virulent C. difficile strains are characterized and identified in diagnostic laboratories by the presence of two potent toxins, TcdA and TcdB (30). These genes are located on a 19.6-kb pathogenicity locus (PaLoc). There is genetic variation in this region which can be exploited and which has revealed 30 different toxinotypes, including six A^−^ B^+^ toxinotypes. The most common and clinically relevant is toxinotype VIII, and these isolates belong to RT017 (31). It is well known that the *tcdA* gene of this type contains a 1.8-kb deletion at the 3′ end and a nonsense mutation at *tcdA* amino acid 47 that introduces a stop codon leading to a truncated *tcdA* gene (31). RT017 strains also lack the binary toxin (CDT) found in, for example, pathogenic RT027 strains that produce all three toxins. Despite lacking two toxins, clinically significant C. difficile infection (CDI) has been reported worldwide for the RT017 lineage (32–41).

Historically, these strains were initially identified in CDI outbreaks in Asia and are thought to have spread to Europe and other continents. RT017 strains have been reported in Canada (35, 42), China (34, 43), South Korea (33, 44, 45), Argentina (46), Australia (47, 48), Israel (49), Japan (50), South Africa (51), and throughout Europe (36, 39, 41, 52, 53). These strains have also been isolated from nonhuman sources, including equines, bovines (54), and rabbits (55). We recently performed WGS on 35 human and two hospital environmental isolates of RT017 circulating in London, United Kingdom, and identified three SNP variants (39). One variant was found to be clonal and had persisted in a London hospital ward for at least 5 years (39).

Here, WGS and phylogenetic analysis were used to define the population structure of a collection of 277 RT017 isolates from six continents of human and nonhuman origins with isolation dates between 1990 and 2013. Analyses reveal that RT017 strains have evolved in parallel from at least two independent sources and can readily transmit between continents. Genotypic and phenotypic antimicrobial susceptibilities were also compared.

## RESULTS

WGS was performed on a global collection of 277 C. difficile RT017 isolates (Table 1). Collectively, these were isolated from human (*n* = 251), bovine (*n* = 9), canine (*n* = 11), equine (*n* = 4), and hospital ward environments (*n* = 2) between 1990 and 2013 (see Information S1 in the supplemental material). All isolates belonged to multilocus sequence type 37. After sequence quality control and mapping to the M68 RT017 reference genome (GenBank accession number FN668375), we identified 1,288 high-quality biallelic SNPs, with 311 present in greater than 1% of samples and greater than 1 bp from an insertion or deletion. Of these non-rare SNPs, 65.6% (*n* = 204) were nonsynonymous, 17.7% (*n* = 55) were synonymous, and 16.7% (*n* = 52) were present in noncoding regions of the genome (nonsynonymous SNPs are shown in Information S2). Twelve SNPs affected stop codons, 11 nonsynonymous and 1 synonymous (Table 1).

**TABLE 1:** Stop codon-associated SNPs

Position in M68 genome	Codon^*a*^		Nonsynonymous/synonymous/noncoding	Gene	Predicted function and/or potential impact	No. of isolates with SNP
	M68 reference	Alternative				
132573	TGG	TGA	Nonsynonymous	M68_00168	Amino acid aminotransferase	16
557896	TTC*	TAA*	Nonsynonymous	*feoB3*	Ferrous iron transport protein B	3
1204039	GGA	TGA	Nonsynonymous	M68_01144	Hydrolase	36
1359584	GGA	TGA	Nonsynonymous	M68_01270	Extracellular solute-binding protein	3
1907433	TAA	GAA	Nonsynonymous	*msrAB*	Peptide methionine sulfoxide reductase	256
1916756	AAT*	GAT*	Synonymous	M68_01782	Unknown	3
3304067	TCA*	GCA*	Nonsynonymous	Sigma-54	Controls expression of nitrogen-related genes	29
3399853	TTG*	TAA*	Nonsynonymous	M68_03193	Ca^2+^/Na^+^ antiporter	13
3402470	CAA	TAA	Nonsynonymous	*plfB*	Formate acetyltransferase	3
3704987	CCA*	TGA*	Nonsynonymous	*sleB*	Spore-cortex-lytic protein	8
3784055	TTC*	TAA*	Nonsynonymous	M68_03513	Penicillin-binding protein	3
4157880	TTG*	TAA*	Nonsynonymous	M68_03851	PTS system, IIc component	6

a *, Located on the reverse strand.

SNP data revealed 109 haplotypes containing between 0 and 52 SNPs (with respect to the M68 reference), with 76.5% (212/277) of isolates having between 10 and 35 SNPs (Table 2).

**TABLE 2:** Summary of details of 277 C. difficile study isolates and their genotypic characteristics

Sublineage	Total no. of isolates	Country of origin	Isolation date	No. (%) of haplotypes	No. of SNPs	No. (%) of isolates with:		Resistance inferred according to position, gene, and aa change^*a*^ [no. (%)]					
						Insertion	Deletion	Rifampin			Fluoroquinolone		
								34,687, *rpoB*, R505K	34,697, *rpoB*, H502N	34,747, *rpoB*, S485F	112,752, *gyrA*, T82I	113,641, *gyrB*, V426D	113,642, *gyrB*, V426I
1	163	Argentina, Australia, Bulgaria, Canada, China, Czech Republic, Greece, Hong Kong, Japan, South Korea, Kuwait, Poland, Portugal, Romania, Singapore, Slovenia, South Africa, The Netherlands, UK, USA	1994 to 2013	55 (50.5)	0–35	49 (30.1)	44 (30)	73 (44.8)	79 (48.5)	0 (0)	124 (76.1)	134 (82.2)	4 (2.5)
2	114	Australia, Hong Kong, Indonesia, Ireland, South Korea, Poland, Singapore, South Africa, Taiwan, The Netherlands, UK, USA	1990 to 2013	54 (49.5)	17–52	65 (57)	109 (96)	17 (15)	13 (11.4)	3 (2.6)	55 (48.2)	114 (100)	9 (7.9)

a Reference residue/amino acid (aa)/alternative residue.

**FIG 1:**
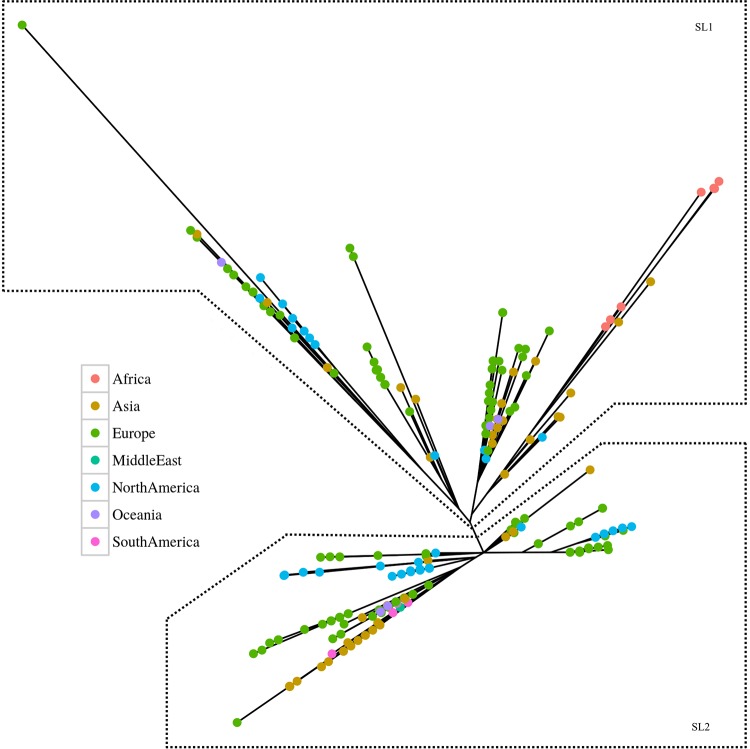
Maximum-likelihood phylogenetic analysis of 277 global RT017 isolates based on core genome SNPs against the M68 reference. We used non-rare (>1% MAF) SNPs that were not in close proximity to insertions or deletions to determine the phylogenic tree. The SL1 and SL2 sublineages were differentiated by four SNPs (Table 3), with the reference strain M68 falling into SL2. The colored nodes indicate the geographical source of isolates.

**FIG 2:**
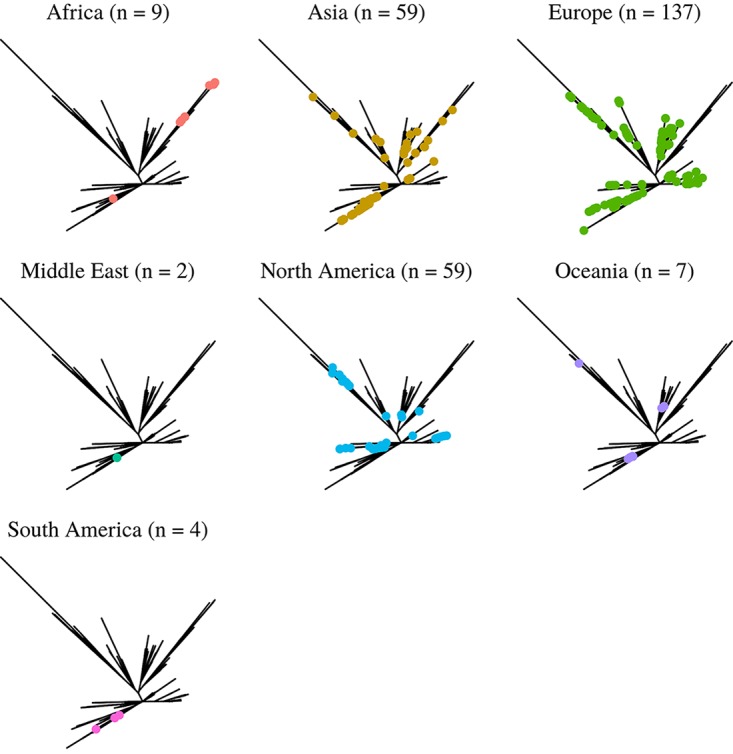
Maximum-likelihood phylogenetic analysis of 277 global RT017 isolates based on core genome SNPs against the M68 reference. The phylogeny is separated into individual panels corresponding to each continent. Data from 5 out of 7 continental designations (Africa, Europe, Asia, Oceania, and North America) include SL1 and SL2 isolates, indicating that both sublineages are global in nature.

We generated a maximum-likelihood phylogenetic tree based on the 1,288 SNPs that demonstrated the presence of two genetically diverse sublineages, SL1 and SL2 (Fig. 1 and 2). Of the 1,288 SNPs, 76% (977/1,288) had a minor allele frequency (MAF) of ≤1% and/or were within 1 bp of an insertion or deletion. To control for false-positive identification of SNPs (these SNPs may mask the true phylogeny of RT017), phylogenetic trees with and without these SNPs were generated. The inclusion of 977 SNPs had a minor effect on the overall phylogenetic tree. Four SNPs were found to differentiate the two sublineages, one present in a noncoding region and three nonsynonymous SNPs (Table 3). SL2 is the most distantly related to the reference M68 strain of the two sublineages, and both sublineages are geographically and temporally widespread. All isolates from the previously reported study on London isolates fell into SL2 (39).

**TABLE 3:** Lineage-defining SNPs

Position	Amino acid	Base		Nonsynonymous/synonymous/noncoding	Gene product	Predicted function and/or potential impact
		Reference	Alternative			
650374	19	A	G	Nonsynonymous	MerR	Altered response to environmental stimuli
900866		C	T	Noncoding		
2914248	257	A	G	Nonsynonymous	DacF	β-Lactam resistance
3604289	329	C	A	Nonsynonymous	Hypothetical protein	Unknown

The RT017 strains are documented to have a higher level of antibiotic resistance than other C. difficile RTs (37, 56). Fluoroquinolone resistance in C. difficile has been associated with mutations in codon 82 of the *gyrA* gene and codon 426 of the *gyrB* gene. The common SNP found in the *gyrA* gene is T82I, and those in the *gyrB* gene are A426V and A426A (57). Remarkably, we found 64.6% (179/277) to have the amino acid substitution found in the *gyrA* gene (T82I). A substitution in the *gyrB* gene (V426N) was present in 4.7% of strains (13/277), and an additional 10.1% (28/277), including M68, harbored a valine at position 426 of the predicted *gyrB* product (Table 2; Information S1). The T82I substitution was globally distributed in both sublineages. Additionally, substitutions in the 81-bp rifampin resistance-determining region of the *rpoB* gene, R505K, H502N, and S485F, were found in 32.5% (90/277), 33.2% (92/277), and 1.1% (3/277) of isolates, respectively (Table 2; Information S1).

**FIG 3:**
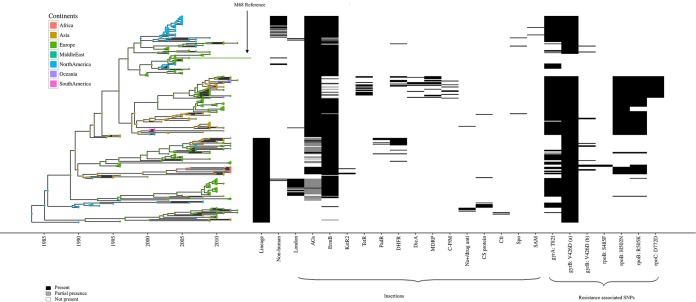
Bayesian evolutionary analysis of 277 global RT017 isolates based on core genome SNPs against the M68 reference. Using a geotemporal model, we can orient the evolution of the RT017 isolates though time. The analysis indicates a split from SL1 (lower) into SL2 (upper) c1990, with the M68 reference in SL2. The introduction of resistance-associated SNPs (such as in *rpoC*) fall within closely related groups in the phylogeny. The continents are colored as described for Fig. 1 and 2. The heat map depicts the sublineage, presence/absence of insertions, and antimicrobial resistance-associated SNPs in relation to the isolates and continent.

To investigate horizontal gene transfer, a key mechanism driving C. difficile evolution, we performed programmatic and visual inspection of the comparisons, which revealed 56 regions of DNA between ∼4 and ∼61.5 kb that were absent from the M68 strain but present in other strains. These had 34 different insertion sites (Table 2 and Fig. 3; Information S1 and S4). Additionally, we found regions of DNA of between ∼8 and ∼29 kb present in the M68 strain at six sites but absent from multiple samples (Table 2; Information S1 and S3). These insertions and deletions were associated with erythromycin, teicoplanin, tetracycline, chloramphenicol, and beta-lactam resistance genes, and their products potentially associated with virulence, such as a two-component response regulator, a SAM protein, an AntA/AntB antirepressor, a cell surface protein, and a sporulation-specific glycosylase (Information S3 and S4). The deletions and insertions were well distributed geographically and temporally, and a 49-kb insertion found only in a clonal cluster of 23/37 London isolates in our previous study (39) was also found to insert at a different site in single isolates from Canada, the United States, and the United Kingdom, with isolation dates of 2006, 2006, and 2011, respectively (Fig. 3). Only one SNP was found in the toxin pathogenicity locus region, which was synonymous and present in the nonfunctioning *tcdA* gene fragment from five South Korean isolates in SL2 isolated between 2004 and 2008. Visual inspection of the comparisons revealed both *tcdA* and *tcdB* genes to be highly conserved; no sequence variations were found.

MICs were determined for eight C. difficile isolates (including M68 as a control) against the antibiotics chloramphenicol, rifampin, tetracycline, erythromycin, nalidixic acid, gentamicin, teicoplanin, and ampicillin. Their MICs are shown in Table 4. All isolates were resistant to nalidixic acid, gentamicin, and ampicillin, were either resistant or intermediately resistant to tetracycline, and were sensitive to teicoplanin. Out of eight isolates, two were resistant to chloramphenicol, four were resistant to rifampin, and seven were resistant to erythromycin.

**TABLE 4:** Antimicrobial susceptibility data and genotypic characteristics

Parameter	Value(s) for strain^*d*^:							
	M68	S-017.72	WA 1514	S-017.92	S-017.27	S-017.74	I6	01-116
Location	Ireland	Walsall, UK	Australia	China	Wrexham, UK	Walsall, UK	Indonesia	South Korea
Yr isolated	2006	2011	2012	2009	1996	2011	2011	2001
Insertion		A, B, C	A		D, E	F, G		
Deletion		H	H, I	J	H, J, K	H, J		
Resistant SNPs								
*rpoB* (R505K)			✓	✓	✓		✓	✓
*rpoB* (H502N)		✓	✓	✓			✓	✓
*rpoB* (S485F)					✓			
*gyrA* (T82I)		✓	✓	✓			✓	
*gyrB* (V426I)					✓			
*gyrB* (V426D)		✓	✓	✓	✓	✓	✓	✓
Antimicrobial agent								
Chloramphenicol^*a*^	8 (S)	8 (S)	4 (S)	64 (R)	8 (S)	8 (S)	256 (R)	8 (S)
Rifampin^*a*^	0.008 (I)	2 (I)	0.004 (S)	>256 (R)	>256 (R)	0.004 (S)	>256 (R)	>256 (R)
Tetracycline^*b*^	32 (R)	32 (R)	0.25 (I)	32 (R)	32 (R)	0.25 (I)	32 (R)	32 (R)
Erythromycin^*b*^	>256 (R)	>256 (R)	>256 (R)	>256 (R)	>256 (R)	<2 (S)	>256 (R)	>256 (R)
Nalidixic acid^*b*^	256 (R)	256 (R)	256 (R)	256 (R)	256 (R)	256 (R)	256 (R)	256 (R)
Gentamicin^*c*^	>256 (R)	>256 (R)	256 (R)	>256 (R)	256 (R)	256 (R)	>256 (R)	>256 (R)
Teicoplanin^*c*^	<1 (S)	<1 (S)	<1 (S)	<1 (S)	<1 (S)	<1 (S)	<1 (S)	<1 (S)
Ampicillin^*b*^	8 (R)	8 (R)	8 (R)	8 (R)	8 (R)	4 (R)	4 (R)	8 (R)

a Recommended by the European Committee on Antimicrobial Susceptibility Testing (EUCAST) (http://www.eucast.org/clinical_breakpoints/).

b Recommended by the CLSI (M11-A8 [58] and M100-S23 [59]).

c No guidance from CLSI or EUCAST. Cutoffs are based on data according to CLSI guideline M100-S23 (interpretative values for Staphylococcus aureus).

d S, sensitive; I, intermediate resistance; R, resistant. Insertions: A, putative drug/sodium antiporter and radical SAM protein TetR-family transcriptional regulator; B, transcriptional repressor DicA; C, streptogramin A acetyltransferase and multidrug resistance protein; D, putative beta-lactamase repressor; E, putative drug/sodium antiporter; F, TetR family transcriptional regulator; G, chloramphenicol o-acetyltransferase (M68 has one copy of chloramphenicol); H, dimethyladenosine transferase (*ermB*); I, putative teicoplanin resistance protein and putative beta-lactamase repressor; J, aminoglycoside 6-adenylyltransferase; K, putative conjugative transposon FtsK_SpoIIIE-related protein.

## DISCUSSION

The RT017 lineage, with its unique toxin profile and unusual global prevalence, has been overshadowed by the global outbreak of the RT027 lineage. Reminiscent of the RT027 lineage, two distinct sublineages of C. difficile RT017 that contain multiple independent clonal expansions were revealed in this study. This division demonstrates that toxin variant strains emerged on at least one occasion, suggesting that a full toxin repertoire is not essential for efficient human-to-human transmission.

Based on our *gyrA* and *gyrB* SNP data, we predict up to 76.2% (211/277) of isolates are resistant to the fluoroquinolone class of antibiotics. Interestingly, the T82I SNP found in *gyrA* is the same mutation reported in the global outbreak of RT027 (4). Based on our MIC data, all eight isolates were resistant to nalidixic acid, indicating resistance to the fluoroquinolone class of antimicrobials.

Based on our rifampin SNP data, we predict 34.7% (96/277) of isolates in this study are resistant to the rifampin class of antibiotics. Interestingly, 82% (152/185) of these substitutions were found in SL1. R505K and H502N have previously been associated with rifampin resistance in C. difficile (60); however, based on our MIC data, only two (2/8) isolates were sensitive to rifampin, with one of the isolates containing the R505K and H502N SNP, indicating that these alone do not always lead to phenotypic resistance. Interestingly, S485F was found in three historical isolates from Wrexham, United Kingdom. This resistance-conferring SNP previously has been reported only in Mycobacterium tuberculosis and not in C. difficile (61). All three isolates were phenotypically resistant to rifampin; however, all three isolates also contained the R505K SNP, confirming this SNP's contribution to resistance was not possible.

The multiple haplotypes revealed is similar to those found for the RT027 global study, where >100 distinct genotypes were found in 151 isolates. Despite SNPs and insertions and deletions, there was no variation in susceptibility to ampicillin, teicoplanin, gentamicin, or nalidixic acid. However, there was some variation with chloramphenicol, rifampin, tetracycline, and erythromycin. Whether the insertions carrying chloramphenicol o-acetyltransferase, the TetR-family transcriptional regulator, or the *ermB* gene played a role in this variation is unknown.

**FIG 4:**
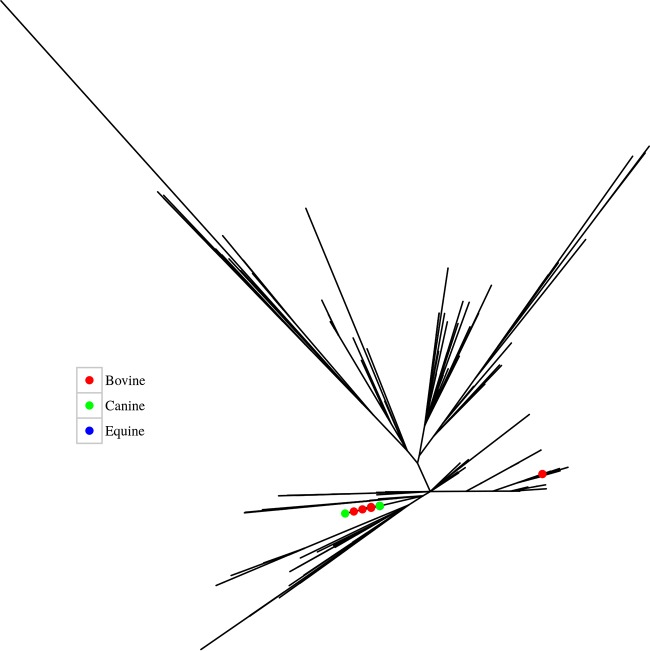
Maximum-likelihood phylogenetic analysis of the global RT017 isolates based on core genome SNPs against the M68 reference depicting the 24 animal isolates by colored nodes. Note the three equine isolates are positioned (and masked) by the bovine and canine cluster on the left. The two bovine isolates on the right of the tree have an SNP distance of 17 from the bovine, canine, and equine cluster. All animal isolates are from Ontario, Canada, and were isolated between 2002 and 2005.

Figure 4 depicts the phylogeny of the isolates by source. Interestingly, the 24 animal strains, which were all isolated from a similar location (Ontario, Canada) over a relatively short time period (2002 and 2005), are distributed among human isolates in SL1 only. This suggests there is transmission between humans and animals.

The ready global distribution of RT017 suggests determinants independent of toxin B are important in transmission. This could be related to the ready acquisition of antibiotic resistance determinants, efficient germination, and/or spore formation. This study provides the basis to further investigate factors important for the epidemic spread of C. difficile.

The deletions and insertions were well distributed geographically and temporally, suggesting either the rapid dissemination of strains or the multiple independent acquisitions and loss of DNA regions (Fig. 2; Information S1). The insertion of different clusters of genes at the same site suggests hot-spot regions for the uptake of DNA (Information S4), and a 49-kb insertion found only in a clonal cluster of 23/37 London isolates in our previous study (39) was also found to insert at a different site in single isolates from Canada, the United States, and the United Kingdom, with isolation dates of 2006, 2006, and 2011, respectively (Fig. 3). This suggests these isolates have independently acquired this insertion.

**FIG 5:**
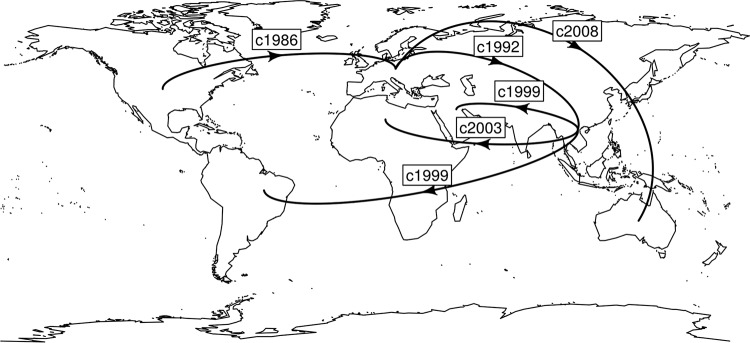
Global transmission events inferred from Bayesian evolutionary analysis of RT017. From the geotemporal analyses we can infer the first movements into each continent, with the date and originating continent. The analysis indicates a North American origin with an expansion into Europe in the mid-1980s, followed by a move into Asia and on to Africa and South America through the 1990s and early 2000s. RT017 was not identified in Oceania (Australia) until the late 2000s, via a jump from Europe.

Similar to RT027, our analyses support a North American origin for RT017 with multiple, global transmission events, with its earliest movement into Europe in 1986 (Fig. 4 and 5). The North American health system and practices appears to facilitate the ready evolution and epidemic spread of C. difficile for RT027 (4) and now, in this study, for RT017. Our data show that it was Europe that introduced RT017 to Asia and Australia, with subsequent spread from Asia to the Middle East, South America, and South Africa. The analysis indicates over 40 movements back and forth over the span of 30 years, consistent with population movements of a globalized society. Traditionally, it has been considered that RT017 strains emerged from Asia due to the reported high incidence of this RT that could not relate to or depend on toxin A-based assays for diagnosis (40). However, our analysis does not support an “out-of-Asia” hypothesis and supports a North American origin (Fig. 4 and 5).

This study investigated the genetic diversity of 277 C. difficile RT017 isolates with temporal, geographical, and source variation. Phylogeographic analysis of the SNPs identified through WGS of the isolates suggests that there are two main sublineages of RT017 that share an ancestry and are globally disseminated. Both sublineages contain isolates from diverse geographical locations and isolation dates, with animal isolates spread among human isolates in SL1. Together with the haplotype diversity and geographically and temporally diverse presence of the transposable elements, these data suggest widespread transcontinental spread and recombination with independent acquisition and loss within different clusters.

## MATERIALS AND METHODS

The 277 isolates described in this study are shown in Table 1 and included 37 isolates from a previous study (ENA study accession number ERP009770) (39), with the remaining being new to this study (ENA study accession number PRJEB11868). These were of human (*n* = 251), environmental/hospital ward (*n* = 2), equine (*n* = 4), canine (*n* = 11), and bovine (*n* = 9) origin, with isolation dates between 1990 and 2013. These isolates were subjected to genomic DNA extraction as previously described by Stabler et al. (62). WGS data for the isolates was obtained using either the HiSeq 2000 sequencing system or the MiSeq sequencing system (Illumina, California), and libraries were created as previously described (63) or using a Nextera XT kit (Illumina, California), respectively. The sequence data were processed and quality controlled according to a standard pipeline as previously described (64). Briefly, FASTQ-formatted sequencing reads were quality controlled with a minimum quality Phred score of 30 (as a rolling average over 4 bases) using trimmomatic (65). The resulting reads were mapped using the BWA-MEM (66) software against the M68 C. difficile reference strain, and the majority of posttrimmed reads (>92% for all samples passing quality control) were mapped to the reference. SNPs were called using SAMtools/VCFtools (67).

Velvet (68) and Velvet Optimizer (http://bioinformatics.net.au/software.velvetoptimiser.shtml) were used to *de novo* assemble the trimmed reads into contigs, producing 277 assemblies. Optimal k-mers fell between 53 bp and 97 bp, and the mean value for median contig size of genome assembly (n50) was over 928,000 bp. The mean longest contig was 1,067,000 bp, with 71 samples producing contigs that covered over half of the genome (greater than ∼2.15 Mbp), and 16 samples assembled to contigs greater than 4 Mbp (equivalent to greater than 90% of the genome). Pipeline, post-, genetic, phylogenetic, phylogeographic, and cluster analyses were carried out using Perl, R, abacas, prokka, RaXML, Bayesian evolutionary analysis sampling trees (BEAST), and mclust software (69–73). A minor allele frequency (MAF) of less than 1% was used. To remove any SNPs that may be associated with recombination and which would mask the true phylogeny, SNPs within 1 bp of an insertion or deletion site were excluded from further analysis. We used BEAST (72) to produce an SNP phylogeny from the SNPs, as well as geographical and temporal data combined in phylogeographic analysis and mclust software for maximum likelihood cluster analysis.

To determine the MICs of 7/277 isolates, dilutions for the antibiotics chloramphenicol, rifampin, tetracycline, erythromycin, nalidixic acid, gentamicin, teicoplanin, and ampicillin were made as previously described (74). Briefly, 10 ml preequilibrated brain heart infusion broth, supplemented with yeast (Oxoid), l-cysteine (Sigma), and C. difficile supplement (Oxoid) (BHIS), were inoculated with three colonies of 48-h culture on BHIS agar plates. Once the optical density (OD) reached 0.3 nm, 24-well plates containing the antibiotic dilutions were inoculated with 1/100 of the BHIS broths and incubated. The ODs were measured 24 h postinoculation, and MIC data were categorized as susceptible, intermediate, and resistant by following the Clinical and Laboratory Standards Institute (CLSI) and the European Committee on Antimicrobial Susceptibility Testing (EUCAST) guidelines. The reference strain M68 was used as a control, as were appropriate negative controls.

### Accession number(s).

Sequence data were deposited in the European Nucleotide Archive under study accession number PRJEB11868.

## Supplementary Material

Supplemental material
