# Supplementary material for: Comparative Genome Analysis and Global Phylogeny of the Toxin Variant Clostridium difficile PCR Ribotype 017 Reveals the Evolution of Two Independent Sublineages
Source: J Clin Microbiol. 2017 Feb 22;55(3):865–76. doi: 10.1128/JCM.01296-16 (PMC5328454; doi:10.1128/JCM.01296-16)
Supplement: Supplemental material [file supp_55_3_865__index.html]

Comparative Genome Analysis and Global Phylogeny of the Toxin Variant Clostridium difficile PCR Ribotype 017 Reveals the Evolution of Two Independent Sublineages — Supplemental material 

# Comparative Genome Analysis and Global Phylogeny of the Toxin Variant Clostridium difficile PCR Ribotype 017 Reveals the Evolution of Two Independent Sublineages

## Supplemental material

- Supplemental file 1 -

  Information S1 (Details of 277 *C. difficile* study isolates and their genotypic characteristics)

  PDF, 498K
- Supplemental file 2 -

  Information S2 (Nonsynonymous SNPs)

  PDF, 167K
- Supplemental file 3 -

  Information S3 (Details of the six deletions)

  PDF, 126K
- Supplemental file 4 -

  Information S4 (Details of the 56 insertions)

  PDF, 188K
- Supplemental file 5 -

  Information S5 (Temporal phylogeny and maximum likelihood clusters C1 to C20 generated by mclust v4 software)

  PDF, 141K
