# Supplementary material for: Comparative Genome Analysis and Global Phylogeny of the Toxin Variant Clostridium difficile PCR Ribotype 017 Reveals the Evolution of Two Independent Sublineages
Source: J Clin Microbiol. 2017 Feb 22;55(3):865–76. doi: 10.1128/JCM.01296-16 (PMC5328454; doi:10.1128/JCM.01296-16)
Supplement: Supplemental material [file JCM.01296-16_zjm999095388s1.pdf]

Supplementary Information 1: Details of 277 *C. difficile* study isolates and their genotypic characteristics. Non-human isolates are indicated by strain names followed by “C” (canine), “B” (bovine), “E” (equine) and “HW” (hospital ward).

Sub-Lineage	Strain Name	Location	Isolation Year	Haplotype	No. of SNPs	Insertion	Deletion	Rifampicin resistance			Fluoroquinolone resistance			Resistance inferred
								34,687	34,697	34,747	112,752	113,641	113,642	Position
								rpoB	*rpoB*	*rpoB*	*gyrA*	*gyrB*	*gyrB*	Gene
								R505K	H502N	S485F	T82I	V426D	V426I	*Amino acid change
1	M04-34	Dublin, Ireland	2004	1	0									
1	M04-21	Dublin, Ireland	2004	1	0									
1	M04-30	Dublin, Ireland	2004	1	0									
1	M04-40	Dublin, Ireland	2004	1	0									
1	M04-50	Dublin, Ireland	2004	1	0									
1	M05-213	Dublin, Ireland	2005	1	0									
1	M05-195	Dublin, Ireland	2005	1	0									
1	M05-214	Dublin, Ireland	2005	1	0									
1	M04-9	Dublin, Ireland	2004	2	2									
1	M08-127	Dublin, Ireland	2008	3	3									
1	M08-150	Dublin, Ireland	2008	3	3									
1	IE 1104	Dublin, Ireland	2009	3	3									
1	SV04-54	Dublin, Ireland	2005	4	5									
1	S3	Dublin, Ireland	2006	4	5									
1	MOH849	Ontario, Canada	2003	5	6						✓			
1	CD257	Ontario, Canada	2003	5	6						✓			
1	CD334	Ontario, Canada	2003	5	6						✓			
1	CD459	Ontario, Canada	2003	5	6						✓			
1	S- 017.8	Dublin, Ireland	2000	6	7									
1	S- 017.9	Dublin, Ireland	2000	6	7									
1	S- 017.10	Dublin, Ireland	2000	6	7									
1	MOH82	Ontario, Canada	2003	7	8						✓			
1	CD656[B]	Ontario, Canada	2004	7	8						✓			
1	CD657[B]	Ontario, Canada	2004	7	8						✓			
1	S- 017.5	Carlisle, UK	1994	8	9	✓						✓		

1	GAI 95601	Japan	1995	8	9							✓	
1	S- 017.13	Japan	1999	8	9							✓	
1	B-33	London, UK	2011	8	9							✓	
1	M04-106	Dublin, Ireland	2005	9	10	✓							
1	M05-185	Dublin, Ireland	2005	9	10								
1	7081082	The Netherlands	2007	10	10	✓						✓	
1	8083571	The Netherlands	2008	10	10							✓	
1	MRL 1135	North Dakota, USA	2007	11	10		✓					✓	
1	UHL-24	London, UK	2013	12	11							✓	
1	MRL 981	California, USA	2007	13	11		✓					✓	✓
1	S- 017.12	Japan	1999	14	11							✓	
1	11-383	Korea	2011	15	11	✓	✓				✓	✓	
1	PT 1307	Portugal	2009	16	13	✓	✓	✓	✓		✓	✓	
1	S- 017.22	Poole, UK	2003	17	13							✓	
1	S- 017.23	Poole, UK	2003	17	13							✓	
1	S- 017.24	Poole, UK	2003	17	13							✓	
1	S- 017.25	Poole, UK	2003	17	13							✓	
1	MRL 529	Michigan, USA	2007	18	13						✓	✓	
1	SV04-9	Dublin, Ireland	2004	19	14	✓							
1	SV04-17	Dublin, Ireland	2004	19	14	✓							
1	SV04-29	Dublin, Ireland	2004	19	14	✓							
1	MRL 532	Michigan, USA	2007	20	15	✓	✓				✓	✓	
1	CD552 [E]	Ontario, Canada	2002	21	15						✓	✓	
1	CD577 [E]	Ontario, Canada	2003	21	15						✓	✓	
1	CD409	Ontario, Canada	2003	21	15						✓	✓	
1	CD159	Ontario, Canada	2003	21	15						✓	✓	
1	CD210	Ontario, Canada	2003	21	15						✓	✓	
1	CD160	Ontario, Canada	2003	21	15						✓	✓	
1	CD425	Ontario, Canada	2003	21	15						✓	✓	
1	CD345	Ontario, Canada	2003	21	15		✓				✓	✓	
1	CD538 [E]	Ontario, Canada	2003	21	15						✓	✓	
1	CD562	Ontario, Canada	2004	21	15						✓	✓	
1	CD744 [B]	Ontario, Canada	2004	21	15						✓	✓	

1	CD472[B]	Ontario, Canada	2004	21	15		✓				✓	✓	
1	CD748[B]	Ontario, Canada	2004	21	15						✓	✓	
1	CD754[B]	Ontario, Canada	2004	21	15						✓	✓	
1	CD755[B]	Ontario, Canada	2004	21	15						✓	✓	
1	CD746[B]	Ontario, Canada	2004	21	15						✓	✓	
1	CD762[B]	Ontario, Canada	2004	21	15						✓	✓	
1	CD479[C]	Ontario, Canada	2005	21	15	✓					✓	✓	
1	CD493[C]	Ontario, Canada	2005	21	15						✓	✓	
1	CD504[C]	Ontario, Canada	2005	21	15						✓	✓	
1	CD515[C]	Ontario, Canada	2005	21	15						✓	✓	
1	CD523[C]	Ontario, Canada	2005	21	15						✓	✓	
1	CD527[C]	Ontario, Canada	2005	21	15						✓	✓	
1	CD528[C]	Ontario, Canada	2005	21	15						✓	✓	
1	CD529[C]	Ontario, Canada	2005	21	15						✓	✓	
1	CD531[C]	Ontario, Canada	2005	21	15						✓	✓	
1	CD578[E]	Ontario, Canada	2005	21	15						✓	✓	
1	CD524[C]	Ontario, Canada	2005	21	15						✓	✓	
1	CD514[C]	Ontario, Canada	2005	21	15						✓	✓	
1	S- 017.15	Kuwait	2001	22	15			✓	✓		✓	✓	
1	S- 017.16	Kuwait	2001	22	15			✓	✓		✓	✓	
1	DS 1383	Singapore	2003	23	16			✓	✓		✓	✓	
1	SI 032	Singapore	2008	23	16			✓	✓		✓	✓	
1	MOH207	Ontario, Canada	2003	24	16	✓					✓	✓	
1	MRL 270	Ontario, Canada	2006	24	16		✓				✓	✓	
1	MRL 102	Ohio, USA	2006	24	16	✓	✓				✓	✓	
1	09-275	Korea	2009	25	16	✓	✓	✓	✓		✓	✓	
1	BG 181	Bulgaria	2009	25	16	✓	✓	✓	✓		✓	✓	
1	GR 0108	Greece	2009	25	16		✓	✓	✓		✓	✓	
1	S- 017.43	Hong Kong	2011	25	16	✓	✓	✓	✓		✓	✓	
1	S- 017.75	Coventry, UK	2011	25	16	✓	✓	✓	✓		✓	✓	
1	S- 017.40	Hong Kong	2011	25	16	✓	✓	✓	✓		✓	✓	
1	SCH5842529	South Africa	2012	25	16	✓	✓	✓	✓		✓	✓	
1	SI 064	Singapore	2012	25	16		✓	✓	✓		✓	✓	

1	WX-36	London, UK	2012	25	16	✓	✓	✓	✓		✓	✓	
1	S- 017.79	Nottinghamshire, UK	2012	25	16	✓	✓	✓	✓		✓	✓	
1	02-210	Korea	2002	27	17		✓	✓	✓		✓	✓	
1	SI 1101	Slovenia	2009	28	17	✓	✓	✓	✓		✓	✓	
1	S- 017.55	Nottinghamshire, UK	2009	28	17	✓	✓	✓	✓		✓	✓	
1	S- 017.91	China	2009	28	17		✓	✓	✓		✓	✓	
1	S- 017.32	Hong Kong	2010	28	17	✓	✓	✓	✓		✓	✓	
1	S- 017.33	Hong Kong	2010	28	17	✓	✓	✓	✓		✓	✓	
1	WX-35	London, UK	2011	28	17	✓	✓	✓	✓		✓	✓	
1	WA 1514	Australia	2012	29	17	✓	✓	✓	✓		✓	✓	
1	MRL 3836	Illinois, USA	2009	30	17	✓	✓				✓	✓	
1	S- 017.77	Coventry, UK	2012	31	18	✓	✓	✓	✓		✓	✓	
1	S- 017.92	China	2009	32	18	✓	✓	✓	✓		✓	✓	
1	CZ 1201	Czech Republic	2009	32	18		✓	✓	✓		✓	✓	
1	675	Romania	2009	32	18		✓	✓	✓		✓	✓	
1	S- 017.20	Poland	2003	35	18							✓	
1	S- 017.1	Argentina	2001	37	18			✓	✓		✓	✓	
1	S- 017.2	Argentina	2001	37	18		✓	✓	✓		✓	✓	
1	S- 017.3	Argentina	2001	37	18			✓	✓		✓	✓	
1	NP-25	London, UK	2008	39	19	✓		✓	✓		✓	✓	
1	ES 531	Australia	2011	39	19	✓	✓	✓	✓		✓	✓	
1	S- 017.35	Hong Kong	2010	40	19	✓	✓	✓	✓		✓	✓	
1	S- 017.37	Hong Kong	2010	40	19	✓	✓	✓	✓		✓	✓	
1	S- 017.38	Hong Kong	2010	40	19	✓	✓	✓	✓		✓	✓	
1	ES 580	Australia	2012	41	19	✓	✓	✓	✓		✓	✓	
1	ES 720	Australia	2012	41	19	✓	✓	✓	✓		✓	✓	
1	12H001405	London, UK	2013	41	19	✓	✓	✓	✓		✓	✓	
1	6797	Argentina	2011	43	19			✓	✓		✓	✓	
1	PL 1204	Poland	2009	49	20							✓	✓
1	01-116	Korea	2001	56	20			✓	✓			✓	
1	S- 017.19	Poland	2003	57	21						✓	✓	
1	6050595	The Netherlands	2006	57	21						✓	✓	
1	7036732	The Netherlands	2007	57	21						✓	✓	

1	S- 017.58	Nottinghamshire, UK	2010	62	22				✓		✓	✓	
1	03-182	Korea	2003	63	22	✓	✓	✓	✓		✓	✓	
1	06-378	Korea	2006	64	22	✓		✓	✓		✓	✓	
1	07-613	Korea	2007	64	22		✓	✓	✓		✓	✓	
1	0 9-02	Korea	2009	64	22	✓		✓	✓		✓	✓	
1	08-191	Korea	2008	65	22	✓	✓	✓	✓		✓	✓	
1	06-472	Korea	2006	66	23	✓		✓	✓		✓	✓	✓
1	07-527	Korea	2007	66	23	✓		✓	✓		✓	✓	✓
1	04-247	Korea	2004	67	23		✓	✓	✓		✓	✓	
1	05-269	Korea	2005	67	23			✓	✓		✓	✓	
1	0 8-26	Korea	2008	67	23			✓	✓		✓	✓	
1	S- 017.48	Coventry, UK	2008	68	23	✓		✓	✓		✓	✓	
1	S- 017.47	Coventry, UK	2008	68	23			✓	✓		✓	✓	
1	S- 017.60	Nottinghamshire, UK	2010	68	23			✓	✓		✓	✓	
1	S- 017.62	Nottinghamshire, UK	2010	68	23			✓	✓		✓	✓	
1	S- 017.68	Coventry, UK	2011	68	23			✓	✓		✓	✓	
1	S- 017.69	Coventry, UK	2011	68	23			✓	✓		✓	✓	
1	S- 017.46	Coventry, UK	2008	72	24			✓	✓		✓	✓	
1	S- 017.50	Coventry, UK	2008	72	24			✓	✓		✓	✓	
1	S- 017.49	Coventry, UK	2008	72	24			✓	✓		✓	✓	
1	S- 017.51	Coventry, UK	2008	72	24			✓	✓		✓	✓	
1	S- 017.52	Coventry, UK	2008	72	24			✓	✓		✓	✓	
1	05-302	Korea	2005	75	25		✓	✓	✓		✓	✓	
1	S- 017.72	Walsall, UK	2011	79	27				✓		✓	✓	
1	S- 017.73	Walsall, UK	2011	79	27				✓		✓	✓	
1	S- 017.64	Walsall, UK	2011	79	27				✓		✓	✓	
1	S- 017.65	Walsall, UK	2011	79	27				✓		✓	✓	
1	S- 017.66	Walsall, UK	2011	79	27				✓		✓	✓	
1	S- 017.90	China	2009	82	30		✓	✓	✓		✓	✓	
1	S- 017.57	Nottinghamshire, UK	2009	83	31			✓	✓		✓	✓	
1	S- 017.59	Nottinghamshire, UK	2010	83	31			✓	✓		✓	✓	
1	S- 017.70	Lincolnshire, UK	2011	84	31	✓	✓	✓	✓		✓	✓	
1	S- 017.86	China	2009	94	34		✓	✓	✓		✓	✓	

1	S- 017.87	China	2009	94	34			✓	✓		✓	✓	
1	S- 017.95	China	2010	94	34			✓	✓		✓	✓	
1	S- 017.88	China	2009	95	35			✓	✓		✓	✓	
1	S- 017.89	China	2009	95	35			✓	✓		✓	✓	
1	S- 017.93	China	2009	95	35		✓	✓	✓		✓	✓	
1	S- 017.94	China	2009	95	35			✓	✓		✓	✓	
2	5992	Illinois, USA	1996	26	17	✓						✓	
2	S- 017.80	Wolverhampton, UK	2012	33	18	✓	✓					✓	
2	S- 017.78	Wolverhampton, UK	2012	33	18	✓	✓					✓	
2	S- 017.36	Hong Kong	2010	34	18	✓	✓					✓	
2	95-24	Korea	1995	36	18	✓	✓					✓	
2	95-25	Korea	1995	36	18	✓	✓					✓	
2	S- 017.14	Japan	1999	38	19	✓						✓	
2	T-14	Taiwan	2011	38	19	✓						✓	
2	MRL 991	California, USA	2007	42	19	✓	✓					✓	
2	QM-34	London, UK	2011	42	19	✓						✓	
2	S- 017.11	Fife, UK	2002	44	19	✓	✓					✓	
2	S- 017.21	Poland	2003	44	19	✓	✓					✓	
2	MRL 738	Washington, USA	2007	44	19	✓	✓					✓	
2	S- 017.30	Hong Kong	2010	44	19		✓					✓	
2	S- 017.31	Hong Kong	2010	44	19	✓	✓					✓	
2	S- 017.41	Hong Kong	2011	44	19		✓					✓	
2	S- 017.45	Hong Kong	2011	44	19	✓	✓					✓	
2	S- 017.44	Hong Kong	2011	44	19	✓	✓					✓	
2	WA 1196	Australia	2012	44	19	✓	✓					✓	
2	S- 017.4	Aylesbury, UK	2005	45	19	✓						✓	✓
2	00-108	Korea	2000	46	19	✓						✓	
2	5572	Minnesota, USA	1992	47	20	✓		✓			✓	✓	
2	6084529	The Netherlands	2006	48	20							✓	
2	SI 010	Singapore	2008	48	20	✓						✓	
2	S- 017.56	Birmingham, UK	2009	50	20	✓						✓	
2	7038446	The Netherlands	2007	51	20	✓					✓	✓	
2	7047343	The Netherlands	2007	51	20	✓	✓				✓	✓	

2	WA 1428	Australia	2012	52	20	✓						✓	
2	SI 004	Singapore	2008	53	20	✓	✓					✓	
2	S- 017.67	Coventry, UK	2011	54	20	✓	✓					✓	
2	S- 017.71	Staffordshire, UK	2011	55	20	✓	✓					✓	✓
2	S- 017.81	Walsall, UK	2012	55	20	✓						✓	✓
2	SI 007	Singapore	2008	58	21			✓	✓			✓	
2	S- 017.76	Coventry, UK	2012	59	21	✓	✓					✓	✓
2	DS 21	Singapore	2003	60	21	✓	✓					✓	
2	I 6	Indonesia	2011	61	22	✓	✓	✓	✓		✓	✓	
2	9029054	The Netherlands	2009	69	23		✓	✓	✓		✓	✓	
2	S- 017.7	Dorchester, UK	1996	70	23	✓						✓	
2	NM-27	Middlesex, UK	2005	71	24	✓					✓	✓	
2	S- 017.6	Dorchester, UK	1996	73	25	✓					✓	✓	
2	4241	Minnesota, USA	1990	74	25	✓						✓	
2	S- 017.53	Worcestershire, UK	2009	74	25	✓						✓	
2	SI 047	Singapore	2012	74	25	✓	✓					✓	
2	SI 099	Singapore	2012	74	25	✓	✓					✓	
2	B-26	London, UK	2009	76	26	✓					✓	✓	
2	S- 017.27	Wrexham, UK	1996	77	26	✓		✓		✓		✓	✓
2	S- 017.28	Wrexham, UK	1996	77	26	✓		✓		✓		✓	✓
2	S- 017.29	Wrexham, UK	1996	77	26	✓		✓		✓		✓	✓
2	T-79	Taiwan	2011	78	27		✓	✓	✓		✓	✓	
2	T-16	Taiwan	2011	78	27		✓	✓	✓		✓	✓	
2	SV04-6	Dublin, Ireland	2004	80	28	✓					✓	✓	
2	SV05-3	Dublin, Ireland	2005	80	28	✓					✓	✓	
2	SV05-25	Dublin, Ireland	2005	80	28	✓					✓	✓	
2	SV04-10	Dublin, Ireland	2004	81	30	✓					✓	✓	
2	SV04-18	Dublin, Ireland	2004	81	30	✓					✓	✓	
2	SV04-19	Dublin, Ireland	2004	81	30	✓					✓	✓	
2	SCH6148880	South Africa	2012	85	31		✓	✓	✓		✓	✓	
2	UHL-4	London, UK	2009	86	31	✓	✓				✓	✓	
2	SCH5806999	South Africa	2012	87	32			✓	✓		✓	✓	
2	SCH6098043	South Africa	2012	87	32			✓	✓		✓	✓	

2	SCH5824693	South Africa	2012	88	32			✓	✓			✓	✓
2	SCH5845556	South Africa	2012	88	32			✓	✓			✓	✓
2	MRL 985	California, USA	2007	89	32	✓						✓	
2	UHL-1	London, UK	2005	90	32	✓	✓				✓	✓	
2	UHL-6	London, UK	2009	90	32	✓	✓				✓	✓	
2	UHL-8	London, UK	2009	90	32	✓	✓				✓	✓	
2	UHL-5	London, UK	2009	90	32	✓	✓				✓	✓	
2	UHL-7	London, UK	2009	90	32	✓	✓				✓	✓	
2	UHL-2	London, UK	2009	90	32	✓	✓				✓	✓	
2	UHL-3	London, UK	2009	90	32	✓	✓				✓	✓	
2	UHL-9	London, UK	2009	90	32	✓	✓				✓	✓	
2	UHL-12	London, UK	2010	90	32						✓	✓	
2	UHL-10	London, UK	2010	90	32	✓	✓				✓	✓	
2	UHL-11	London, UK	2010	90	32	✓	✓				✓	✓	
2	UHL-13	London, UK	2010	90	32	✓					✓	✓	
2	UHL-14	London, UK	2010	90	32	✓					✓	✓	
2	UHL-22	London, UK	2010	90	32	✓	✓				✓	✓	
2	SI 006	Singapore	2008	91	33	✓	✓					✓	
2	CDP08WTH7	Liverpool, UK	2010	91	33	✓						✓	
2	UHL-15	London, UK	2010	92	33	✓	✓				✓	✓	
2	UHL-16	London, UK	2010	92	33	✓	✓				✓	✓	
2	UHL-17	London, UK	2010	92	33	✓	✓				✓	✓	
2	UHL-21	London, UK	2010	92	33	✓	✓				✓	✓	
2	UHL-23	London, UK	2011	92	33	✓	✓				✓	✓	
2	UHL-19 [HW]	London, UK	2010	93	34	✓	✓				✓	✓	
2	UHL-21 [HW]	London, UK	2010	93	34	✓	✓				✓	✓	
2	UHL-18	London, UK	2010	93	34	✓	✓				✓	✓	
2	MRL 2923	Colorado, USA	2008	96	36	✓	✓					✓	
2	5733	Illinois, USA	1995	97	37	✓	✓				✓	✓	
2	8070899	The Netherlands	2008	98	37	✓	✓					✓	
2	CX-32	London, UK	2011	98	37	✓						✓	
2	MRL 2259	California, USA	2008	99	38	✓						✓	
2	4092	Minnesota, USA	1990	100	38	✓	✓				✓	✓	

2	4139	Minnesota, USA	1990	100	38	✓	✓				✓	✓	
2	H-219	Illinois, USA	2006	100	38	✓	✓				✓	✓	
2	12H400159	London, UK	2013	101	39	✓	✓				✓	✓	
2	5264	Minnesota, USA	1992	102	39	✓	✓				✓	✓	
2	5265	Minnesota, USA	1992	102	39	✓	✓				✓	✓	
2	5340	Minnesota, USA	1993	102	39	✓	✓				✓	✓	
2	S- 017.84	Northamptonshire, UK	2012	103	39	✓					✓	✓	
2	S- 017.82	Northamptonshire, UK	2012	103	39	✓					✓	✓	
2	S- 017.83	Northamptonshire, UK	2012	103	39	✓					✓	✓	
2	GOSH-28	London, UK	2010	104	41	✓						✓	
2	GOSH-29	London, UK	2010	105	47	✓						✓	
2	GOSH-30	London, UK	2010	105	47	✓						✓	
2	RF-31	London, UK	2010	105	47	✓	✓					✓	
2	S- 017.61	Birmingham, UK	2010	106	48	✓	✓					✓	
2	S- 017.74	Walsall, UK	2011	106	48	✓	✓					✓	
2	S- 017.85	Northamptonshire, UK	2012	106	48	✓	✓					✓	
2	WA 0908	Australia	2012	106	48	✓	✓					✓	
2	GOSH-37	London, UK	2013	107	49	✓						✓	
2	SCH5865760	South Africa	2012	108	51			✓	✓			✓	
2	SCH5864722	South Africa	2012	109	52		✓	✓	✓		✓	✓	
2	SCH6163235	South Africa	2012	109	52			✓	✓		✓	✓	

* Reference Residue/Amino Acid/ Alternative Residue
