# Supplementary material for: Comparative Genome Analysis and Global Phylogeny of the Toxin Variant Clostridium difficile PCR Ribotype 017 Reveals the Evolution of Two Independent Sublineages
Source: J Clin Microbiol. 2017 Feb 22;55(3):865–76. doi: 10.1128/JCM.01296-16 (PMC5328454; doi:10.1128/JCM.01296-16)
Supplement: Supplemental material [file JCM.01296-16_zjm999095388s2.pdf]

Supplementary Information 2: The 204 non-synonymous SNPs and their predicted function and impact.

Position	Reference Base	Alternative Base	Amino Acid	Gene/Predicted Function and/or Potential Impact	Number of isolates with SNP
23868	G	T	6	*rpsJ* (tigecyline resistance)	265
1907433	T	G	282	*msrAB* (altered response to environmental stress)	256
113641	A	T	426	*gyrB* (fluoroquinolone resistance)	248
288057	C	A	56	*phnM* (degradation of phosphonate compounds)	248
1869520	A	G	144	*hisC* (histidinol-phosphate aminotransferase)	248
112752	G	A	82	*gyrA* (fluoroquinolone resistance)	179
650374	A	G	19	*MerR* (altered response to environmental stimuli)	114
2914248	A	G	257	*dacF* (Beta-lactam resistance)	114
3604289	C	A	329	Hypothetical protein	114
34697	G	T	502	*rpoB* (rifampicin resistance)	92
34687	C	T	505	*rpoB* (rifampicin resistance)	90
1918864	T	C	292	*bioB* (altered biotin production, limiting its availability and in-turn increasing toxin synthesis)	59
3419885	A	C	314	Signalling protein	59
800885	C	T	20	PTS system, IIa component (altered sensitivity to bacteriocins)	55
72976	C	T	218	Beta-glucosidase (hydrolysis of cellobiose to two molecules of glucose)	39
3355379	C	A	346	Signalling protein	39
1204039	G	T	141	HAD superfamily hydrolase (hydrolytic enzyme reactions)	36
213561	C	T	193	Hypothetical protein	29
345335	A	C	31	Protein-tyrosine-phosphatase (control of the biosynthesis of capsular polysaccharides and extracellular polysaccharides)	29
465423	T	C	97	Hypothetical protein	29
1666328	T	C	87	Signalling protein	29
1669112	G	T	392	Signalling protein	29
2155937	G	A	134	Glyoxalase (altered response to environmental stress)	29

2761655	T	C	104	MerR (altered response to environmental stimuli)	29
3304067	T	G	559	Sigma-54 (controls expression of nitrogen related genes)	29
3366100	G	T	164	Permease (membrane transporter)	29
400308	C	A	321	*cbiK* (iron transport system)	26
2764775	C	T	676	Signalling protein	26
3067079	C	G	497	Penicillin-binding protein (B-lactam resistance)	26
4025381	G	A	11	*FlgG* (altered formation of flagella)	26
3066391	G	A	726	Penicillin-binding protein (B-lactam resistance)	25
3202066	T	A	332	ABC transporter, permease/ATP-binding protein	25
578215	C	T	252	Iron-only hydrogenase	23
707105	C	A	98	ABC transporter, permease protein	23
1123155	G	A	125	Membrane protein	23
1541265	G	A	394	Sensor histidine kinase	23
2236997	C	T	429	*aroA*	23
3072208	C	G	66	*maf*	23
3403871	G	T	115	*plfA*	23
2881638	T	G	223	*tepA*	20
457651	C	A	37	Multidrug efflux protein	19
1178734	G	T	223	Cell surface protein	19
1942550	T	A	158	Signalling protein	19
1997615	A	T	133	Xanthine/uracile permease	19
3053095	C	T	162	*obg*	19
3364434	G	A	227	CoA-transferase	19
3916418	G	A	239	Hypothetical protein	19
3511614	T	C	9	Transporter	18
132573	G	A	330	Amino acid aminotransferase	16
850525	C	T	163	*bglG1*	16

2764219	G	T	491	Signalling protein	16
1032720	C	T	275	ABC transporter, substrate-binding protein	15
2060217	G	A	67	*argC*	15
2075525	G	T	854	*clpB*	15
2800650	C	A	356	Oxidoreductase	15
3382194	G	A	555	Penicillin-binding protein (B-lactam resistance)	15
4024527	G	A	31	*flgG*	15
113642	C	T	426	*gyrB*	13
2284466	G	T	174	Nitrite and sulfite reductase subunit	13
2733799	G	A	55	*folP*	13
3399853	G	A	310	Ca2+/Na+ antiporter	13
4184694	T	A	197	*buk*	13
50281	C	A	317	Dual-specificity prolyl/cysteinyl-tRNA synthetase	12
1115288	G	A	48	Aromatic amino acid aminotransferase	12
1303022	G	A	9	Lipoprotein	12
1394996	C	A	145	TetR-family trancscriptional regulator	12
3066199	A	G	790	Penicillin-binding protein (B-lactam resistance)	12
3066407	A	G	721	Penicillin-binding protein (B-lactam resistance)	12
2764414	G	A	556	Diguanylate kinase signaling protein	11
3066235	G	A	778	Penicillin-binding protein (B-lactam resistance)	11
1382195	A	G	641	Exported protein	10
1977841	G	T	212	Xanthine/uracil permease	10
2961596	C	T	498	*smc*	10
3174341	A	C	90	Cell surface protein (Putative N-acetylmuramoyl-L-alanine amidase)	10
3174343	T	C	89	Cell surface protein (Putative N-acetylmuramoyl-L-alanine amidase)	10
388057	G	T	323	*cbiP*	9
470760	G	C	264	*bclA3*	9

585426	C	T	50	Peptidase	9
657360	G	T	515	Sensor histidine kinase	9
1048935	G	A	67	Drug/sodium antiporter	9
1276180	C	A	78	Hypothetical protein	9
1381396	A	G	375	Exported protein	9
1458207	C	T	358	Bifunctional protein	9
1474488	C	T	364	Sodium: dicarboxylate symporter family protein	9
1538843	T	C	249	*selB*	9
2495508	G	A	81	Exported protein	9
2646577	A	G	339	Iron-sulfur protein	9
2656639	C	T	691	*feoB2*	9
2902717	T	C	473	*acd*	9
3066331	G	A	746	Penicillin-binding protein (B-lactam resistance)	9
3166570	C	T	449	Helicase	9
3229965	C	A	675	Two-component sensor histidine kinase	9
3300923	C	A	281	PTS system lactose/cellobiose-family transporter subunit IIC	9
3514262	G	A	302	Transposase-like protein b	9
3514307	A	G	287	Transposase-like protein b	9
3514802	C	T	122	Transposase-like protein b	9
3514805	G	A	121	Transposase-like protein b	9
567449	G	T	632	Two-component sensor histidine kinase	8
1252965	A	C	86	Two-component response regulator	8
1491685	T	C	27	*kstR2*	8
1949814	T	G	265	Membrane protein	8
3239437	G	A	337	*leuC*	8
3382096	A	G	522	Penicillin-binding protein (B-lactam resistance)	8
3704987	C	T	19	*sleB*	8

3751894	T	G	1315	Cell surface protein (Putative hemagglutinin/adhesin) precursor	8
644128	C	T	259	Two-component sensor histidine kinase	7
2629377	C	A	475	Signalling protein	7
2636947	A	G	631	ABC transporter, permease protein	7
382360	T	G	171	Aldose epimerase00416	6
682915	G	A	305	*xdhA3*	6
763280	C	T	152	Hypothetical protein	6
839287	C	T	148	Transcription antiterminator	6
1165584	G	T	537	*secA2*	6
1828018	C	A	289	Signalling protein	6
4157880	G	A	395	PTS system, IIc component	6
15593	C	T	20	*rpsE*	5
51607	C	A	487	*proS*	5
221342	C	T	274	*pyrAB1_2*	5
232498	C	T	200	Hypothetical protein	5
383181	C	A	26	Hypothetical protein	5
395438	A	G	3	*cbiT*	5
850279	C	T	81	*bglG1*	5
906912	G	A	39	*glvC*	5
939561	A	G	61	Hypothetical protein	5
1208476	A	G	478	Signalling protein	5
1242643	A	T	4	Aminotransferase	5
1303484	G	A	163	Lipoprotein	5
1508773	C	A	18	Two-component sensor histidine kinase	5
1694380	G	A	221	Oligoendopeptidase	5
1698282	G	A	77	*grdE*	5
1927579	G	T	59	Serine/threonine protein kinase	5

3066400	G	A	723	Penicillin-binding protein (B-lactam resistance)	5
3147022	G	A	117	Phosphoesterase	5
3267110	A	G	24	*speE*	5
51218	A	T	4	Dual-specificity prolyl/cysteinyl-tRNA synthetase	4
127314	A	G	47	*soj_1*	4
1873506	G	T	199	Acetyl-CoA synthetase	4
2438942	G	T	158	Lipoprotein	4
2444939	C	T	143	Iron compound ABC transporter, permease protein	4
2830244	A	T	256	Phage protein	4
2933682	C	A	225	*topA*	4
3581263	G	C	496	*hrsA_2*	4
3784489	C	T	373	Penicillin-binding protein (B-lactam resistance)	4
3850253	A	G	86	Hypothetical protein	4
34747	G	A	485	*rpoB* (rifampicin resistance)	3
464168	G	A	11	Transcriptional regulator	3
511931	C	T	43	*hydN1*	3
557896	C	A	342	*feoB3*	3
569182	C	T	181	Hypothetical protein	3
612296	C	A	21	*bclA2_1*	3
660220	T	C	285	ABC transporter (salivaricin lantibiotic)	3
714135	G	A	112	Hypothetical protein	3
918006	T	C	299	Hypothetical protein	3
941032	A	G	96	Thioredoxin	3
941686	C	T	287	Aaminotransferase	3
996373	G	A	212	ABC transporter, substrate-binding protein	3
1050326	C	T	709	Signalling protein	3
1064970	A	C	394	Sigma-54-dependent transcriptional regulator	3

1161805	T	C	74	*slpA*	3
1162228	A	G	215	*slpA*	3
1185215	C	T	451	*pgm2*	3
1206681	G	A	259	N-acetylmuramoyl-L-alanine amidase"	3
1220630	C	T	417	Radical SAM family protein	3
1250085	C	T	138	Transferase	3
1359584	G	T	272	Extracellular solute-binding protein	3
1405479	T	C	247	Carbon starvation	3
1463838	G	A	207	Sugar transporter, permease protein	3
1553544	G	C	104	Lipoprotein	3
1795359	G	A	343	PTS system, IIbc component	3
1907960	G	T	89	Hypothetical protein	3
1920960	G	T	8	ATP/GTP-binding protein	3
1932695	G	A	43	Membrane protein	3
2036515	T	C	212	Hypothetical protein	3
2060944	C	T	309	*argC*	3
2084143	T	G	96	TetR-family transcriptional regulator	3
2373940	A	G	159	Molybdopterin-guanine biosynthesis protein	3
2401429	G	T	101	Membrane protein	3
2457775	C	T	580	Calcium-transporting ATPase	3
2523615	G	A	142	Arylesterase	3
2704311	C	T	180	Ruberythrin	3
2719354	G	A	143	ABC transporter, ATP-binding protein	3
2764540	G	A	598	Signalling protein	3
2801003	G	A	239	Oxidoreductase	3
2943677	G	A	66	Hypothetical protein	3
2987909	C	A	103	Penicillin-binding protein (B-lactam resistance)	3

3036429	G	A	47	*fabH*	3
3067082	T	C	496	Penicillin-binding protein (B-lactam resistance)	3
3253744	G	T	343	*opuCC*	3
3306641	T	A	136	*oppF*	3
3340579	A	C	78	Oxidative stress regulatory protein	3
3382086	T	G	519	Penicillin-binding protein (B-lactam resistance)	3
3402470	C	T	445	*plfB*	3
3522538	G	A	135	RNA polymerase sigma factor	3
3626504	T	G	222	Two-component sensor histidine kinase	3
3627605	T	A	160	Hypothetical protein	3
3660789	G	A	144	Chemosensory protein	3
3784055	C	A	518	Penicillin-binding protein	3
3831154	T	C	51	DNA-binding transcriptional activator YeiL	3
3832857	G	A	63	Acetyltransferase	3
3924724	G	T	628	ABC transporter, permease protein	3
4000580	T	C	5	ABC transporter, permease protein	3
4130077	G	A	76	Amidohydrolase/peptidase	3
4169330	G	A	37	*fabZ*	3
4180249	C	T	148	Phosphoglucomutase/phosphomannomutase mutase	3
