# Supplementary material for: Comparative Genome Analysis and Global Phylogeny of the Toxin Variant Clostridium difficile PCR Ribotype 017 Reveals the Evolution of Two Independent Sublineages
Source: J Clin Microbiol. 2017 Feb 22;55(3):865–76. doi: 10.1128/JCM.01296-16 (PMC5328454; doi:10.1128/JCM.01296-16)
Supplement: Supplemental material [file JCM.01296-16_zjm999095388s3.pdf]

Supplementary Information 3: Details of the six deletions and their corresponding genes of interest and putative function.

Deletion Site in M68	Size (bps)	No. of isolates	Sub-Lineage	No. of CDS	Genes of Interest/Putative Function
480308	27,982	67 (24.2%)	SL1 and SL2	31	DNA-directed RNA polymerase sigma-70 factor, sporulation initiation inhibitor, dimethyladenosine transferase (ermB), zeta toxin, antitoxin epsilon, omega transcriptional repressor, ABC transporter, ATP-binding protein, GntR-family transcriptional regulator, cell wall hydrolase, DNA-binding protein, collagen-binding surface protein
2172459	27,985	8 (2.9%)	SL1	18	Aminotransferase, aminobenzoyl-glutamate transport protein, peptidase, transcriptional regulator, bacterial alpha-L-rhamnosidase, xylose transporter, AraC-family transcriptional regulator, ethanolamine/propanediol utilization protein, two-component sensor histidine kinase, two-component response regulator
2210949	8,866	8 (2.9%)	SL1	10	Membrane protein, ABC transporter, anti-sigma factor, ECF family, RNA polymerase sigma factor, carbohydrate acetyl esterase/feruloyl esterase precursor, PadR family transcriptional regulator, AraC-family transcriptional regulator, site-specific recombinase
2527343	9,890	99 (35.7%)	SL1 and SL2	7	Acetyltransferase, bifunctional AAC/APH, aminoglycoside 6-adenylyltransferase
2969461	16,713	5 (1.8%)	SL1 and SL2	15	RNA polymerase sigma-E factor, AIPR protein, teicoplanin resistance protein, beta-lactamase repressor, membrane protein, cell surface protein, lipoprotein
3934113	22,222	28 (10.1%)	SL2	15	DNA-binding protein, translation elongation factor G, cell wall hydrolase, collagen-binding surface protein.
