# Supplementary material for: Comparative Genome Analysis and Global Phylogeny of the Toxin Variant Clostridium difficile PCR Ribotype 017 Reveals the Evolution of Two Independent Sublineages
Source: J Clin Microbiol. 2017 Feb 22;55(3):865–76. doi: 10.1128/JCM.01296-16 (PMC5328454; doi:10.1128/JCM.01296-16)
Supplement: Supplemental material [file JCM.01296-16_zjm999095388s4.pdf]

Supplementary Information 4: Details of the 56 Insertions and their genes of interest and putative function.

Insertion Site in M68	Size (bps)	Hot-Spot Region	No. of isolates	Sub-Lineage	No. of CDS	Genes of Interest/Putative Function
163761	20,710	No	2	SL2	8	Radical SAM enzyme, Cfr family, antirestriction protein ArdA, PrgI family protein
421208	11,349	No	1	SL2	13	AraC-family transcriptional regulator, energy-coupling factor transporter transmembrane protein EcfT, ABC transporter, ATP-binding/permease protein, drug/sodium antiporter, sigma factor,
428266	39,503	No	2	SL2	35	Membrane protein, replicative DNA helicase, PrgI family protein, cell surface protein, DNA topoisomerase, AraC-family transcriptional regulator, energy-coupling factor transporter transmembrane protein EcfT, ABC transporter, ATP-binding protein, drug/sodium antiporter, sigma factor
446832	39,563	No	19	SL2	12	Stage 0 sporulation protein J, Phd_YefM, PrgI family protein, modification methylase MboII, sortase B, cell wall hydrolase, iron-sulfur protein, two-component response regulator, RNA polymerase sigma factor, regulator of cell growth
480452	39,420	No	4	SL1	12	Stage 0 sporulation protein J, Phd_YefM, PrgI family protein, modification methylase MboII, sortase B, cell wall hydrolase, iron-sulfur protein, two-component response regulator, RNA polymerase sigma factor, regulator of cell growth
470354	27,999	No	8	SL1 and SL2	6	Modification methylase DpnIIB, cell surface protein, DNA topoisomerase, Antirestriction protein (ArdA), Serine/threonine-protein kinase and phosphatase

480414	39,420	Yes	3	SL1	18	Radical SAM protein, RNA polymerase sigma-24 subunit, ECF subfamily, protease HtpX, helicase, DNA topoisomerase, cell surface protein, cell wall hydrolase, PrgI family protein, Stage 0 sporulation protein J, sporulation initiation inhibitor
480414	25,515	Yes	1	SL1	10	Stage 0 sporulation protein J, PrgI family protein, cell wall hydrolase, cell surface protein, SnoaL-like domain protein, Group II intron-encoded protein LtrA, DNA topoisomerase
480414	42,282	Yes	1	SL1	16	soj_2 spo0J_2, cell surface protein, SnoaL-like domain protein, Group II intron-encoded protein LtrA, DNA topoisomerase, helicase, MerR-family transcriptional regulator, D-beta-hydroxybutyrate dehydrogenase, Putative NADPH-dependent FMN reductase, Protease HtpX
480414	39,501	Yes	2	SL1	13	Sporulation initiation inhibitor, cell surface protein, DNA topoisomerase, helicase, protease HtpX, RNA polymerase, sigma-24 subunit, ECF subfamily, radical SAM protein
533968	7,875	No	3	SL2	8	Helix-turn-helix domain protein, ABC transporter, ATP-binding/permease protein, HTH-type transcriptional repressor KstR2, replication initiation factor
568265	32,213	Yes	2	SL2	14	Helix-turn-helix domain protein, Arc-like DNA binding domain protein, YqaJ-like viral recombinase domain protein, recombination and repair protein RecT, YopX protein, endodeoxyribonuclease RusA, NB-ARC domain protein, AIPR protein putative regulatory protein, beta-lactamase repressor
568265	6,588	Yes	1	SL1	7	YcfA-like protein, beta-lactamase repressor
612300	25,542	No	1	SL2	8	PrgI family protein, bacterial mobilization protein (MobC)

612311	29,943	No	2	SL2	24	PrgI family protein, DNA methylase, Serine/threonine-protein kinase and phosphatase
654816	10,449	No	1	SL1	11	Helix-turn-helix domain protein, drug/sodium antiporter, radical SAM protein, TetR-family trancscriptional regulator, glycosyl transferase family 2, integrase
697659	10,260	No	15	SL1	12	Helix-turn-helix domain protein, drug/sodium antiporter, radical SAM protein, TetR-family trancscriptional regulator, glycosyl transferase family 2, Integrase
815847	12,708	No	1	SL2	4	SAP domain protein, ribbon-helix-helix protein, copG family, beta-lactamase repressor
820852	9,270	Yes	6	SL2	9	Bifunctional AAC/APH, transcriptional regulator PadR-like family protein
820852	7,209	Yes	1	SL1	7	Bifunctional AAC/APH
820852	8,118	Yes	1	SL1	7	Riboflavin biosynthesis protein
918087	10,343	Yes	2	SL1 and SL2	15	Integrase, helix-turn-helix domain protein, ribbon-helix-helix protein, copG family, nickel-responsive regulator

918087	8,046	Yes	1	SL2	5	Ribbon-helix-helix protein, copG family, YcfA-like protein, integrase
918087	19,610	Yes	2	SL2	8	Arc-like DNA binding domain protein, AntA/AntB antirepressor, siphovirus Gp157, ERF superfamily protein, YopX protein, endodeoxyribonuclease RusA, NB-ARC domain protein
918087	53,351	Yes	2	SL1 and SL2	10	Ribbon-helix-helix protein, copG family, nickel-responsive regulator, helix-turn-helix domain protein, siphovirus Gp157, ERF superfamily protein, YopX protein, Modification methylase DpnIIA, endodeoxyribonuclease RusA
1032928	6,354	Yes	1	SL1	8	ABC transporter, ATP-binding protein, Helix-turn-helix domain protein, siphovirus Gp157
1032928	49,281	Yes	1	SL1	14	ABC transporter, ATP-binding protein, siphovirus Gp157, ERF superfamily protein, YopX protein, Endodeoxyribonuclease RusA, AntA/AntB antirepressor, putative kinase, haemolysin XhlA, holin
1298645	11,385	Yes	1	SL1	14	Integrase, nickel-responsive regulator, AIPR protein, beta-lactamase repressor
1298645	9,810	Yes	1	SL1	6	Integrase, nickel-responsive regulator, Helix-turn-helix domain protein, siphovirus Gp157 beta-lactamase repressor
1298645	6,291	Yes	1	SL1	9	Integrase, YcfA-like protein, ribbon-helix-helix protein, copG family, Arc-like DNA binding domain protein, AntA/AntB antirepressor, siphovirus Gp157, ERF superfamily protein

1298645	10,035	Yes	1	SL1	5	nickel-responsive regulator, Arc-like DNA binding domain protein, helix-turn-helix domain protein, siphovirus Gp157, ERF superfamily protein
2203180	7,911	No	22	SL1 and SL2	6	Bacterial transcription activator, effector binding domain 5-amino-6-(5-phosphoribosylamino) uracil reductase, dihydrofolate reductase region, DNA-binding protein
2253920	18,549	No	1	SL2	8	Cell surface protein, DNA topoisomerase, bacterial mobilization protein (MobC), Integrase
2551711	22,734	No	2	SL1 and SL2	7	Terminase-like family protein
2579949	33,021	No	1	SL1	11	Kinase, AntA/AntB antirepressor, endodeoxyribonuclease RusA, peptide chain release factor 2, helix-turn-helix domain protein, YopX protein, ERF superfamily protein, siphovirus Gp157, ABC transporter ATP-binding protein
2633741	9,297	No	1	SL2	8	TetR-family trancscriptional regulator, radical SAM protein, helix-turn-helix domain protein
3316864	8,380	Yes	7	SL1	4	Transcriptional repressor DicA
3316864	8,487	Yes	1	SL1	6	Multidrug transporter MatE, marR, MobA/MobL family protein,

3316864	8,433	Yes	1	SL1	6	MobA/MobL family protein, helix-turn-helix domain protein, transcriptional repressor DicA
3764961	17,631	No	3	SL1	13	Integrase, helix-turn-helix domain protein, ABC transporter, ATP-binding/permease protein, RNA polymerase, sigma-24 subunit, ECF subfamily, SAM protein, collagen-binding surface protein
3835852	61,155	No	1	SL2	24	Dimethyladenosine transferase (ermB), streptogramin A acetyltransferase, antibiotic resistance ABC transporter, ATP-binding protein, multidrug resistance protein, N-acetyl-anhydromuranmyl-L-alanine amidase, holin, sporulation-specific glycosylase YdhD, ATP-dependent Clp protease proteolytic subunit, AIG2-like family protein, amidoligase enzyme, BsuMI modification methylase subunit YdiP, DNA adenine methyltransferase YhdJ, S-adenosylmethionine synthetase, HNH endonuclease, phosphoribosylformimino-5-aminoimidazole carboxamide ribotide isomerase, virulence-associated protein e, DNA-directed dna polymerase, restriction enzyme subunit
3854778	6,571	No	1	SL2	4	Chloramphenicol o-acetyltransferase, resolvase family
3873711	32,589	Yes	14	SL1	22	Transcriptional regulator, corrin/porphyrin methyltransferase, 16S rRNA (guanine(1405)-N(7))-methyltransferase, replicative dna helicase, bacterial mobilization protein (MobC), (alpha)-aspartyl dipeptidase, dimethyladenosine transferase, transcriptional regulator, Maff2 family protein, AraC-family transcriptional regulator, streptogramin A acetyltransferase, antibiotic resistance ABC transporter, multidrug resistance protein
3873711	12,771	Yes	6	SL1	12	Corrin/porphyrin methyltransferase
3876204	11,700	No	1	SL1	5	Modification methylase HhaI, Bsp6I restriction endonuclease, resolvase family putative

3879955	12,555	No	5	SL1 and SL2	13	Drug/sodium antiporter, ABC transporter, ATP-binding/permease protein, energy-coupling factor transporter transmembrane protein EcfT, AraC-family transcriptional regulator, transcriptional regulator
4002688	13,932	Yes	1	SL2	8	Virulence-associated protein e, two-component system response regulator, two-component sensor histidine kinase, ABC transporter, ATP-binding protein
4002688	12,800	Yes	1	SL2	6	lantibiotic ABC transporter, ATP-binding protein, sensor histidine kinase, salavaricin, two-component response regulator
4002696	9,171	Yes	2	SL2	5	Transcriptional regulator PadR-like family protein, bifunctional AAC/APH, DNA-binding protein
4002696	6,282	Yes	1	SL1 and SL2	6	Bacterial transcription activator, effector binding domain, 5-amino-6-(5-phosphoribosylamino)uracil reductase, Group II intron-encoded protein LtrA, dihydrofolate reductase region, DNA-binding protein
4002696	8,613	Yes	1	SL2	9	Riboflavin biosynthesis protein
4002696	10,395	Yes	1	SL1	11	Sensor protein, two-component system, response regulator, ABC transporter, permease protein, ATP-binding protein
4092174	4,230	No	3	SL1 and SL2	8	Beta-lactamase repressor, regulatory protein, helix-turn-helix domain protein

3879948	9,342	Yes	1	SL2	7	Regulator of cell growth, RNA polymerase sigma factor RpoE, two-component sensor histidine kinase, ABC-2 family transporter protein, ATP-binding protein, two-component response regulator
3879948	40,774	Yes	2	SL2	18	RNA polymerase sigma factor RpoE, two-component sensor histidine kinase, ABC-2 family transporter protein, ATP-binding protein, two-component response regulator, regulatory protein, cell wall hydrolase, Putative sortase B, PrgI family protein, helicase, Cna protein B-type domain protein, stage 0 sporulation protein, sporulation initiation inhibitor
4305005	54,494	No	1	SL2	16	Helix-turn-helix domain protein, siphovirus Gp157, ERF superfamily protein, YopX protein, modification methylase DpnIIA, Endodeoxyribonuclease RusA, terminase-like family protein
