# Supplementary material for: Comparative Genome Analysis and Global Phylogeny of the Toxin Variant Clostridium difficile PCR Ribotype 017 Reveals the Evolution of Two Independent Sublineages
Source: J Clin Microbiol. 2017 Feb 22;55(3):865–76. doi: 10.1128/JCM.01296-16 (PMC5328454; doi:10.1128/JCM.01296-16)
Supplement: Supplemental material [file JCM.01296-16_zjm999095388s5.pdf]

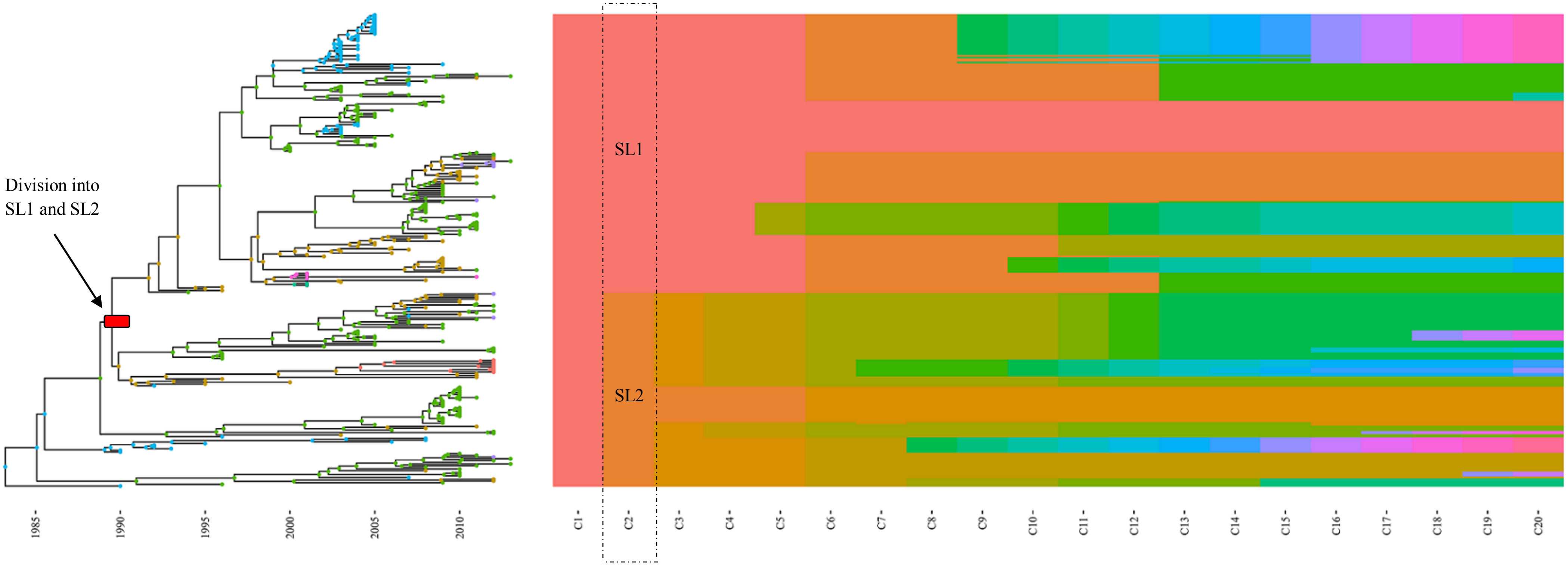

Supplementary Information 5: Temporal phylogeny and maximum likelihood clusters C1 to C20 generated by mclust v4 software. Like in figure 4, the phylogenetic tree illustrates how the collection of isolates has evolved over time with continents coloured accordingly and sub-lineage depicted accordingly. The heat map on this figure splits the collection of 277 isolates into 20 possible clusters based on maximum likelihood generated by mclust software. Cluster 2 (C2) was selected as the best fit based on epidemiological, genetic and temporal data associated with the isolates.
